# Prevalence and predictors for sustained remission in rheumatoid arthritis

**DOI:** 10.1371/journal.pone.0214981

**Published:** 2019-04-19

**Authors:** Yoon-Kyoung Sung, Kazuki Yoshida, Femke H. M. Prince, Michelle L. Frits, Soo-Kyung Cho, Jung-Yoon Choe, Hye-Soon Lee, Jisoo Lee, Shin-Seok Lee, Dae-Hyun Yoo, Simon M. Helfgott, Nancy A. Shadick, Michael E. Weinblatt, Daniel H. Solomon, Sang-Cheol Bae

**Affiliations:** 1 Division of Rheumatology, Brigham and Women’s Hospital, Boston, Massachusetts, United States America; 2 Department of Rheumatology, Hanyang University Hospital for Rheumatic Diseases, Seoul, South Korea; 3 Department of Paediatrics/ Paediatric Rheumatology, Erasmus MC Sophia Children’s Hospital, Rotterdam, The Netherlands; 4 Department of Rheumatology, Catholic University of Daegu School of Medicine, Daegu, South Korea; 5 Department of Rheumatology, Hanyang University Guri Hospital, Guri, South Korea; 6 Division of Rheumatology, Ewha Womans University Mokdong Hospital, Seoul, South Korea; 7 Department of Rheumatology, Chonnam National University Hospital, Gwangju, South Korea; Universite de Nantes, FRANCE

## Abstract

**Objective:**

Remission is a key goal in managing rheumatoid arthritis (RA), with sustained remission as the preferred sequelae of short-term remission. However little is known about the predictors of sustained remission for patients reaching remission. Using two independent cohorts, we aimed to evaluate the prevalence and predictors for sustained remission.

**Methods:**

The study cohort consisted of subjects with RA from the Brigham and Women's Hospital Rheumatoid Arthritis Sequential Study (BRASS) and the Korean Observational Study Network for Arthritis (KORONA). We analyzed subjects who reached remission in 2009 with follow up data for two consecutive years. Remission was defined by the Disease Activity Score 28- (DAS28-CRP) of less than 2.6. Sustained remission was defined as three consecutive annual visits in remission. Predictors for sustained remission were identified by multivariate logistic regression analysis.

**Results:**

A total of 465 subjects were in remission in 2009. Sustained remission was achieved by 53 of 92 (57.5%) in BRASS and by 198 of 373 (53.1%) in KORONA. In multivariate analyses, baseline predictors of sustained remission were: disease duration less than 5 years [odds ratio (OR) 1.96, 95% confidence interval (95% CI) 1.08–3.58], Modified Health Assessment Questionnaire (MHAQ) score of 0 (OR 1.80, 95% CI 1.18–2.74), and non-use of oral glucocorticoid (OR 1.58, 95% CI 1.01–2.47).

**Conclusion:**

More than half of RA subjects in remission in 2009 remained in remission through 2011. Short disease duration, no disability, and non-use of oral glucocorticoid at baseline were associated with sustained remission.

## Introduction

Rheumatoid arthritis (RA) is a chronic, systemic inflammatory disease that affects synovial joints. The pathology of the disease process often leads to serious functional disability and chronic pain caused by destruction of the joint [1]. Previous studies have reported that intensive management of RA improves radiographic disease progression, physical function, and quality of life [2–5]. Hence, remission is a key goal that is now achievable with advances in available therapies [6, 7]. Since disease progression is more likely to occur in patients achieving short-term remission than in those achieving sustained remission, which is more likely to improve patient outcomes [7]. However, a small number of patients can achieve sustained remission in clinical practice [8], and disease flare and recurrence can happen unexpectedly, even if patients achieve remission [3]. Therefore, identifying the predictors of sustained remission in clinical practice is important for physicians and patients to plan treatments.

Until now, several studies identifying predictors for sustained remission have been conducted, and they suggest that male sex, younger age, short duration of symptoms, few tender joints count, low functional disability, low disease activity or radiologic score, and a good response to initial treatment at baseline are independent predictors [9–13]. However, various definitions of remission in RA have been used and the rates of remission have been reported differently according to criteria [14–16]. In addition, definitions of sustained remission varied across studies, and there are only a few studies that have assessed a remission longer than one year. Most prior studies focused on early RA, even though most RA patients in clinical practice have suffered from the disease for a long time.

In this study, we aimed to investigate the prevalence of sustained remission in established RA patients, and to identify the predictors for this.

## Patients and methods

### Study design and data sources

We combined data from two large RA cohorts. The Brigham and Women’s Hospital Rheumatoid Arthritis Sequential Study (BRASS) is a single-center, prospective, observational cohort of over 1,300 RA patients [17]. Enrollment for the registry began in March of 2003 in the Brigham and Women’s Hospital Arthritis Center in the United States. At the baseline visit and at each annual follow up visit, patient’s rheumatologists complete forms detailing extra-articular manifestations, comorbidities, medication changes, laboratory test results, 28-joint count and overall disease activity score. A trained staff member interviews the patient and the patient fills out a self-administered questionnaire containing questions about medication and patient-reported outcomes. Patient questionnaires are completed every six months.

The KORean Observational study Network for Arthritis (KORONA) is a multicenter, prospective, observational cohort of Korean RA patients. It was established in 2009 by the Clinical Research Center for Rheumatoid Arthritis in Hanyang University funded by the ministry of health and welfare, South Korea [18]. All RA patients aged 18 or older who satisfied the 1987 American College of Rheumatology criteria for RA and who planned to have their blood drawn for routine evaluation were asked to provide informed consent. Of these, more than 95% of patients consented to participate in the KORONA cohort [18]. All enrolled patients completed an initial questionnaire to establish their demographic profile, medical history, and disease-specific outcomes. Disease activity with joint assessments were made by patient’s rheumatologists or well-trained medical staffs. These evaluations, together with laboratory test results, prescription information, and patient self-evaluations are integrated in the database and they are completed with annual follow-up. A total of 5,300 RA patients from 23 South Korean centers have been included in KORONA.

### Study participants

Both cohorts have a similar study design and subject inclusion criteria: patients with established RA diagnosed by rheumatologists, with virtually all patients aged over 18 years at their enrollment. However, since differences in initial enrollment periods for each cohort can influence drug availability, we restricted this study to include subjects who had an annual visit from 2009 through 2011 (n = 382 in BRASS and n = 1,290 in KORONA). Among them, a total of 1171 patients (188 in BRASS and 983 in KORONA) who had 3 years of disease activity data were selected for complete case analysis. For the analysis of prevalence and predictors for sustained remission lasting two consecutive years, we further selected patients who were in remission in 2009 (S1 Fig). Each patient signs an informed consent form that was obtained according to the Declaration of Helsinki and approved by the Partners Institutional Review Board (IRB) at Brigham and Women’s Hospital and IRB at Hanyang University Hospital (HYUH 2009-04-003). The current analytic protocol was approved by the Partners Institutional Review Board (IRB) at Brigham and Women’s Hospital (# 2012P-00096).

### Sustained remission

Disease activity status was determined using disease activity score (DAS) 28 based on C reactive protein (CRP) scores [19]. DAS28-CRP was calculated using four variables [DAS28-CRP(4)]: swollen and tender joint counts, CRP, and patient global assessment. Remission was defined by DAS28-CRP(4) remission (score < 2.6) primarily; we also assessed rates of remission by the American College of Rheumatology (ACR)/European League Against Rheumatism (EULAR) remission criteria. We included in this study patients achieving DAS28-CRP(4) remission in 2009. Patients were classified as being in “sustained remission” if they were in DAS28-CRP(4) remission at 2 consecutive annual visits after baseline. We conducted a nested case-control study of patients with or without sustained remission.

### Statistical analysis

We used descriptive statistics to characterize baseline patient demographics and disease characteristics in each cohort separately and in aggregate. The duration of remission was characterized for all subjects in remission in 2009, examining the annual assessments in 2010 and 2011. The rates of sustained remission were compared across cohorts and bivariable relationships between sustained remission and demographic features such as age, sex, body mass index (BMI), income, and education level as well as clinical features such as disease duration, presence of disability, medication such as disease modifying antirheumatic drugs (DMARDs), non-steroidal anti-inflammatory drugs (NSAIDs), pain killer, glucocorticoid, anti-osteoporotic medication and autoantibody positivity were examined.

Variables significant at the P<0.25 level in the univariate model were advanced to multivariate logistic regression models to determine predictors of sustained remission. Predictors and parameter estimates of each cohort were very similar. Thus, we fit a model combining cohorts with an indicator term for cohort (BRASS or KORONA)

All statistical analyses were conducted using SAS System for Windows version 9.2 (SAS Institute Inc., Cary, N.C.) and SPSS version 18.0 (SPSS Inc, Chicago IL). Results were considered statistically significant when *P* values were less than 0.05.

## Results

### Demographic and clinical characteristics

Among the subjects who had 3 years follow up data of disease activity (n = 1,171), the point prevalence of DAS28-CRP(4) remission in 2009 was 39.7% in total (49.2% in BRASS and 37.9% in KORONA), whereas ACR/EULAR remission in 2009 was only 8.5% (6.4% in BRASS and 8.7% in KORONA) (S1 Fig). A total of 465 RA subjects from BRASS (n = 92) and KORONA (n = 373) with remission according to DAS28-CRP(4) were selected for our study. The demographic and clinical characteristics at baseline are summarized by each cohort in Table 1. The proportion of females among total subjects was 81.9% and the mean age was 55.5±11.7 years. Although there was no difference in age (P = 0.78) and sex (P = 0.91) between two cohorts, BRASS subjects had longer disease duration, higher income and education level, and higher BMI compared to KORONA. Subcutaneous nodules were more common among BRASS subjects, whereas rheumatoid factor (RF) and anti-citrullinated protein antibody (ACPA) were more common among KORONA subjects (91.7% and 89.0% in KORONA subjects compared to 62.7% and 60.9% in BRASS subjects, P < 0.01). Composite measures of disease activity were slightly worse among BRASS subjects than KORONA, but functional disability measured with modified health assessment questionnaire score (MHAQ) and the number of co-morbidity were similar between two cohorts.

**Table 1:** Baseline characteristics of the study population (in 2009).

Individual characteristics	Total (n = 465)	BRASS (n = 92)	KORONA (n = 373)	P
emographic feature				
Age in years, mean (SD)*	55.5 (11.7)	55.2 (11.1)	55.6 (11.9)	0.624
Female	381 (81.9)	75 (81.5)	306 (82.0)	0.507
Education level				<0.001
~ High school	300 (64.8)	52 (28.0)	277 (74.3)	
College ~	163 (35.2)	134 (72.0)	96 (25.7)	
NA	2	2	0	
Income ($/year)				<0.001
~9,999	121 (26.3)	1 (1.1)	120 (32.3)	
10,00	216 (47.0)	11 (12.4)	205 (55.3)	
50,000~89,999	56 (12.2)	17 (19.1)	39 (10.5)	
90,000~	67 (14.6)	60 (67.4)	7 (1.9)	
NA	5	3	2	
Body mass index, mean (SD)* kg/m^2^	23.1 (3.9)	25.9 (5.3)	22.4(3.1)	<0.001
Clinical features				
Years with RA, mean (SD)*	9.2 (8.1)	14.3 (9.0)	7.9 (7.3)	0.001
Tender joint count, mean (SD)*	0.3 (0.7)	0.3(0.7)	0.4 (0.7)	0.870
Swollen joint count, mean (SD)*	1.1 (1.3)	1.0 (1.9)	0.3(0.8)	<0.001
Rheumatoid nodule	27 (5.8)	12 (13.3)	15 (4.0)	<0.001
Modified HAQ, mean (SD)*	0.14 (0.30)	0.14 (0.30)	0.14 (0.31)	0,796
DAS28-CRP(4), mean (SD)*	1.9 (0.4)	1.7 (0.4)	2.0 (0.4)	0.016
SDAI, mean (SD)*	5.1 (2.5)	3.9 (3.1)	5.4 (2.8)	0.359
CDAI, mean (SD)*	4.8 (2.9)	3.8 (3.1)	5.0 (2.8)	0.338
Rheumatoid factor positivity	394/456 (86.4)	52/83(62.7)	342 (91.7)	<0.001
ACPA positivity	322/391(82.4)	56 (60.9)	266/299 (89.0)	<0.001
Charlson Comorbidity Index, mean (SD)*	0.37 (0.65)	0.3(0.6)	0.4 (0.7)	0.138
ACR/EULAR remission	98 (21.1)	12 (13.0)	86 (23.1)	0.021
Treatment				
Biologics DMARD	73 (15.7)	52 (56.5)	21 (5.6)	<0.001
Non-biologic DMARD	438 (94.2)	68 (73.9)	967 (98.4)	<0.001
Methotrexate	393 (84.5)	58 (63.0)	335 (89.8)	<0.001
Methotrexate dose, mean (SD)* mg/week	13.3 (4.0)	17.2 (6.1)	12.6 (3.0)	<0.001
Leflunomide	166 (35.8)	4 (4.3)	162 (43.5)	<0.001
Non-steroidal anti-inflammatory drug.	193 (41.5)	44 (47.8)	149 (39.9)	0.105
Cyclooxygenase-2 inhibitor	98 (21.1)	8 (8.7)	90 (24.1)	<0.001
Acetaminophen	37 (8.0)	20 (21.7)	17 (4.6)	<0.001
Opioid	14 (3.0)	3(3.3)	11(2.9)	0.508
Anti-osteoporotic medication	149 (32.0)	59 (64.1)	90 (24.1)	<0.001
Glucocorticoid	286 (61.6)	17 (18.7)	269 (72.1)	<0.001
Glucocorticoid dose, mean (SD)* mg/day	4.0 (1.9)	2.3 (1.4)	4.2 (1.8)	0.250

* Except where indicated otherwise, values are the number (%). BRASS, Brigham Rheumatoid Arthritis Sequential Study; KORONA, KORean Observational study Network for Arthritis; RA, rheumatoid arthritis; SD, standard deviation; NA, not available; ACPA, anti-citrullinated protein antibody; HAQ, health assessment questionnaire; DAS28-CRP(4), disease activity score 28-C-reactive protein using 4 variables; CDAI, clinical Disease Activity; SDAI, Simplified Disease Activity Index; ACR, American College of Rheumatology; EULAR, European League Against Rheumatism; DMARD, disease modifying anti-rheumatic drug.

Patterns of medication use for subjects in remission were quite different between the two cohorts. Biologic disease modifying anti-rheumatic drugs (DMARDs) were more commonly used in BRASS, whereas methotrexate and glucocorticoid were commonly used in KORONA.

### Prevalence of sustained remission

Two hundred and fifty-one (21.6%) subjects who had 3 years follow up data (n = 1,171) achieved sustained remission from 2009 to 2011. The prevalence of DAS28-CRP(4) sustained remission for subjects with remission in 2009 (n = 465) was 54.0%. The prevalence of DAS28-CRP(4)sustained remission was similar for BRASS (57.5%) and KORONA (53.1%) (P = 0.44, Table 2A). The prevalence of ACR/EULAR sustained remission for subjects with remission in 2009 (n = 99) was only 17 (17.2%) with no significant difference between BRASS and KORONA (P = 0.13, Table 2B)

**Table 2:** Prevalence of sustained remission among patients with remission in 2009.

**A. Sustained remission according to DAS 28-CRP(4)**							
Remission	2009	2010	2011	Total (n, %)	BRASS (n, %)	KORONA (n, %)	*P*
Sustained^**^	**R**	**R**	**R**	251 (54.0)	53 (57.5)	198 (53.1)	0.44^*^
**Not sustained**	**R**	**R**	A	86 (18.5)	17 (18.5)	69 (18.5)	
	**R**	A	**R**	68 (14.6)	11 (12.0)	57 (15.3)	
	**R**	A	A	60 (12.9)	11 (12.0)	49 (13.1)	
Patients with remission in 2009				465 (100)	92 (100)	373 (100)	
**B. Sustained remission according to ACR /EULAR remission criteria**							
Sustained^**^	**R**	**R**	**R**	17 (17.2)	4 (33.3)	13 (14.9)	0.13^*^
**Not sustained**	**R**	**R**	A	13 (13.1)	3 (25.0)	10 (11.5)	
	**R**	A	**R**	18 (18.2)	1 (8.3)	17 (19.5)	
	**R**	A	A	51 (51.5)	4 (33.3)	47 (54.0)	
Patients with remission in 2009				99 (100)	12 (100)	87 (100)	

^*^sustained remission or not in patients with remission in 2009

^**^ sustained remission from 2009 to 2011; BRASS, Brigham Rheumatoid Arthritis Sequential Study; KORONA, KORean Observational study Network for Arthritis; ACR, American College of Rheumatology; EULAR, European League Against Rheumatism; R. remission; A, active.

### Predictors for sustained remission

In the unadjusted logistic regression model with DAS28-CRP(4) sustained remission as the outcome (Table 3), variables associated with sustained remission included: younger age (odds ratio [OR] 2.53, 95% confidence interval [95% CI] 1.42–4.53 for less than 45 years and OR 1.70, 95% CI 1.08–2.67 for 45–64 years group), shorter disease duration (OR 1.92, 95% CI 1.14–3.24 for less than 5 years and OR 1.72, 95% CI 1.00–2.95 for 5–9 years group), and higher education level (OR 1.70, 95% CI 1.15–2.50). In addition, MHAQ of 0 (OR 2.27, 95% CI 1.54–3.36) non-use of oral glucocorticoid (OR 1.49, 95% CI 1.03–2.17) at baseline were associated with sustained remission.

**Table 3:** Predictors for sustained remission in rheumatoid arthritis patients (n = 465).

Variables	Univariate analysis	Multivariate analysis		
		*P*	OR	CI
Cohort				
BRASS (n = 92)	1.20 (0.76–1.90)	0.85	1.06	(0.58–1.95)
Age (years)				
65 ~ (n = 88)	1	0.31	1	
45 ~ 64 (n = 270)	1.70 (1.08–2.67)*	0.25	1.33	(0.82–2.17)
~ 44 (n = 107)	2.53 (1.42–4.53)*	0.13	1.66	(0.86–3.21)
Years with RA				
15 ~ (n = 165/463)	1	0.16	1	
10 ~ 14.9 (n = 137/463)	1.77 (0.94–3.31)	0.08	1.82	(0.93–3.55)
5 ~ 9.9 (n = 73/463)	1.72 (1.00–2.95)*	0.09	1.68	(0.93–3.04)
~ 4.9 (n = 88/463)	1.92 (1.14–3.24)*	0.03	1.96	(1.08–3.58)**
Education level				
~ High school diploma (n = 300/463)	1		1	
College ~ (n = 163/463)	1.70 (1.15–2.50)*	0.22	1.34	(0.84–2.15)
Sex				
Male (n = 84)	1.40 (0.86–2.26)	0.30	1.21	(0.78–1.86)
Modified HAQ				
Score = 0 (no disability) (n = 299/459)	2.27 (1.54–3.36) *	0.01	1.80	(1.18–2.74) **
Glucocorticoid use				
No (n = 278)	1.49 (1.03–2.17) *	0.05	1.58	(1.01–2.47) **
Charson comorbidity index				
No (n = 329)	1.42 (0.95–2.12)	0.39	1.21	(0.78–1.87)
Rheumatoid factor				
Negative (n = 394/456)	1.40 (0.81–2.42)	0.44	1.27	(0.69–2.35)
Anti-citrullinated protein antibody				
Negative (n = 69/391)	1.04 (0.62–1.75)			
Income ($/year)				
~9,999 (n = 121/460)	1			
10,000~49,999 (n = 216/460)	1.35 (0.86–2.76)			
50,000~89,999 (n = 56/460)	1.87 (0.98–3.58)			
90,000~ (n = 67/460)	1.47 (0.81–2.68)			
Obesity (Body mass index)				
Underweight ~18.4 (n = 29/462)	0.68 (0.31–1.47)			
Normal 18.5~24.9 (~22.9 in WPRO) (n = 248/462)	1			
Pre-obese 25~29.9 (23~24.9 in WPRO) (n = 94/462)	1.18 (0.73–1.89)			
Obese 30~ (25~ in WPRO) (n = 91/462)	0.93 (0.58–1.50)			
Biologic DMARD				
Yes (n = 73)	0.91 (0.55–1.51)			
Non-biologic DMARD				
Yes (n = 438)	0.80 (0.36–1.75)			
Methotrexate use				
Yes (n = 393)	0.76 (0.46–1.26)			
Non-steroidal anti-inflammatory drug use				
Yes (n = 193)	1.07 (0.74–1.55)			

^*^P<0.05 in univariate analysis

^**^ P<0.05 in multivariate analysis; OR, odds ratio; CI, 95% confidence interval; BRASS, Brigham Rheumatoid Arthritis Sequential Study; HAQ, health assessment questionnaire; WPRO, world health organization western pacific region office; DMARD, disease-modifying anti-rheumatic drug

In a multivariate model predicting sustained remission, we included all of the above significant variables and those with p < 0.25 in unadjusted regression. As shown in Table 3, disease duration less than 5 years (OR 1.96, 95% CI 1.08–3.58), MHAQ of 0 (OR 1.80, 95% CI 1.18–2.74), and non-use of glucocorticoid (OR 1.58, 95% CI 1.01–2.47) were significantly associated with increased odds for sustained remission in RA. Cohort (BRASS or KORONA) was not a predictor for sustained remission in multivariate model. The multivariate models for each cohort were presented in S1 Table.

## Discussion

The present study investigated the prevalence and predictors for sustained remission in established RA. The long-term follow-up data from the BRASS and KORONA cohorts revealed that sustained remission occurred during 2009 through 2011 in 54.0% of patients with remission in 2009. Disease duration less than 5 years, MHAQ of 0, and non-use of oral glucocorticoid were predictors of sustained remission in multivariate analysis.

Most studies about predictors for sustained remission have been reported in early RA cohorts [9, 11, 12, 20], but few studies have been performed in established RA. In early RA cohorts, low disease activity at baseline [9, 12, 20], short symptom duration [11, 12], male [12, 20], younger age [20], the absence of autoantibodies [11], and lesser radiologic score at baseline [9] were all predictors for sustained remission. However, our study using established RA cohort did not find the association of sex, age, and the negativity of RF with sustained remission. Until now, only two studies of established RA exist from the Consortium of Rheumatology Researchers of North America (CORRONA) database [21, 22]. One study examined early and established disease cohorts separately and found that male sex is the major predictor for sustained remission in early diseases but not in established RA. Conversely, the use of glucocorticoid and disease activity at baseline were inversely associated with sustained remission in established RA patients [22]. Our results were similar to the results of the previous CORRONA study in which they show that the baseline characteristics that are indicative of less severe or well controlled disease might be associated with sustained remission and sex was not a predictor for sustained remission in established RA patients. While the present study is similar to this prior work, it is difficult to compare the results of two studies because our study was different in several regards. First, the definition of sustained remission in the prior work was the remission of 2 consecutive visits after baseline that were more than 2 months, and up to 6 months, apart. Our definition of sustained remission required 3 visits over 2 years in remission. Second, disease activity measure was clinical disease activity index (CDAI) in the previous study and DAS28-CRP(4) for our study. Third, variables used in the predictive models varied.

As other studies have demonstrated, patients with shorter RA duration are more likely to achieve remission [23]. In our study, shorter RA duration and MHAQ of 0 were identified as predictors of sustained remission in established RA patients. This finding supports the concept of a therapeutic window of opportunity early in the disease course and indicates that early intervention that induce remission before development of functional disability may be critical for sustained remission. Non-use of oral glucocorticoid was also associated with sustained remission. However, further study should be performed to identify whether it is from the glucocorticoid use or other confounders, such as biologic agents or the severity of the underlying disease

Our study has several important strengths. First, our definition of sustained remission was over 2 years in remission and it is longer than previous studies. Second, our study was performed with established RA patients. Although it is difficult to achieve remission in these patients, we can try to taper DMARDs. Therefore, this information regarding sustained remission in established RA patients can be used as the basis for tapering DMARDs therapy.

We acknowledge the limitations of our work. First, we restricted the population to the same period from the two cohorts which were established in different time periods. This may cause selection bias in the BRASS cohort because it was established before KORONA, although it allows us to compare the two cohorts with similar drug availability. According to a previous report, patients with a shorter disease duration, higher disease activity, and several other socioeconomic factors were associated with the loss to follow-up from the BRASS cohort [24]. In addition, we used listwise deletion as a method for handling missing data, because these data go missing completely at random. Although this may cause selection bias, we did not perform data imputation because our outcome for regression analysis was remission based on disease activity. Furthermore, because both cohorts enrolled patients with RA regardless of their disease duration, we could not confirm at enrollment whether or not the patients were in remission for the first time. Second, the patients were assessed for remission according to DAS28-CRP(4) criteria (<2.6). Although a previous study reported the tendency for DAS28-CRP(4) to underestimate DAS28-ESR(4) and recommended that the thresholds for remission should be changed to 2.3 in using DAS28-CRP(4), this only has a marginal improvement over the original cut-off of 2.6 for DAS28-CRP(4) in another study [25]. For the strict criteria for remission, although there is the ACR/EULAR remission criteria, we decided to use the DAS28-CRP(4) remission criteria because this is more commonly used in clinical practice and its psychometric properties are excellent [26]. However, since it is more liberal in defining remission, it allowed us to identify additional predictors for sustained remission. Third, we have assumed that the levels of disease activity evaluated at the 1 year time point represents a stable period in a particular disease activity state, whereas in reality, patients may have experienced limited fiares during that time. Although this ‘area under the curve’ approach to evaluate longitudinal disease activity would be ideal, it is difficult in the setting of standard clinical care. Hence we regarded sustained remission as staying in a remission state for 3 consecutive annual visits during a 2 year time period to ensure that the disease activity period was stable. Fourth, we assembled two cohorts from different countries, and while the baseline characteristics differed, the predictors of sustained remission were similar and the cohort was not a significant in adjusted models. An interesting observation from our results is that the proportion of sustained remission in patients with remission in 2009 in each cohort was not different. However, we could not assess the drug adherence (which can affect the remission rate in each cohort) although we did assess the several treatments such as DMARDs, pain killers and glucocorticoids. Lastly, the point prevalence of remission in 2009 is quite similar to that obtained for the groups in the BeSt study (38–46%), but higher than our previous study [18] and other observational studies [21, 27]. However, this might be from the inclusion period used in our study. Since we used recent data between 2009 and 2011, the remission rate would be higher than before due to the availability of new treatment options and guidelines.

## Conclusions

Our study demonstrated that the 2 year sustained remission rate of subjects in remission in 2009 was 54%. We identified several predictors of sustained remission that might be useful in stratifying patients with established RA in typical practice. Such variables may help identify patients who can attempt discontinuation of DMARDs or whose therapy should be closely monitored since they are a great risk of flares after reaching remission.

## Supporting information

S1 Fig Patient selection flow and cohort assembly of BRASS and KORONA. (DOCX)

S1 Table Multivariate logistic regression analysis on predictors for sustained remission in each cohort. (DOCX)
