# Correction: Prevalence and predictors for sustained remission in rheumatoid arthritis

**DOI:** 10.1371/journal.pone.0221314

**Published:** 2019-08-14

**Authors:** Yoon-Kyoung Sung, Kazuki Yoshida, Femke H. M. Prince, Michelle L. Frits, Soo-Kyung Cho, Jung-Yoon Choe, Hye-Soon Lee, Jisoo Lee, Shin-Seok Lee, Dae-Hyun Yoo, Simon M. Helfgott, Nancy A. Shadick, Michael E. Weinblatt, Daniel H. Solomon, Sang-Cheol Bae

The email address listed for the corresponding author, Yoon-Kyoung Sung, is incorrect. The correct email address for the corresponding author is: sungyk@hanyang.ac.kr.
