# Supplementary material for: Prevalence and predictors for sustained remission in rheumatoid arthritis
Source: PLoS One. 2019 Apr 19;14(4):e0214981. doi: 10.1371/journal.pone.0214981 (PMC6474583; doi:10.1371/journal.pone.0214981)
Supplement: S1 Fig — (DOCX) [file pone.0214981.s001.docx]

BRASS

Cohort

KORONA

Patients with visit in 2009

3,155

618

Patients with 3 year follow-up during 2009 and 2011

1,290

382

Patients with 3year data of disease activity including DAS 28-CRP(4) and

ACR /EULAR remission criteria

1,171 (BRASS 188, KORONA 983)

Patients with remission in 2009 (%)

ACR/EULAR remission

98 (8.4)

DAS 28-CRP(4) remission

465 (39.7)

S1 Fig. Patient selection flow and cohort assembly of BRASS and KORONA.

* Values are number otherwise indicated. BRASS, Brigham Rheumatoid Arthritis Sequential Study; KORONA, KORean Observational study Network for Arthritis; DAS28, Disease Activity Score 28-C-reactive protein; ACR, American College of Rheumatology; EULAR, European League Against Rheumatism.
