# Supplementary material for: Prevalence and predictors for sustained remission in rheumatoid arthritis
Source: PLoS One. 2019 Apr 19;14(4):e0214981. doi: 10.1371/journal.pone.0214981 (PMC6474583; doi:10.1371/journal.pone.0214981)
Supplement: S1 Table — (DOCX) [file pone.0214981.s002.docx]

S1 Table. Multivariate logistic regression analysis on predictors for sustained remission in each cohort

| Variables | Total | | |  | BRASS | | |  | KORONA | | |
| --- | --- | --- | --- | --- | --- | --- | --- | --- | --- | --- | --- |
|  | P | OR | 95% CI |  | P | OR | 95% CI |  | P | OR | 95% CI |
| Cohort  BRASS | 0.85 | 1.06 | (0.58-1.95) |  |  |  |  |  |  |  |  |
| Age (years)  65 ~  45 ~ 64  ~ 44 | 0.31  0.25  0.13 | 1  1.33  1.66 | (0.82-2.17)  (0.86-3.21) |  | 0.81  0.52  0.76 | 1  0.65  0.76 | (0.18-2.36)  (0.13-4.44) |  | 0.12  0.09  0.05 | 1  1.62  2.13 | (0.93-2.82)  (1.00-4.51) ^*^ |
| RA duration (years)  15 ~  10 ~ 14.9  5 ~ 9.9  ~ 4.9 | 0.16  0.08  0.09  0.03 | 1  1.82  1.68  1.96 | (0.93-3.55)  (0.93-3.04)  (1.08-3.58)^*^ |  | 0.11  0.04  0.04  0.47 | 1  5.07  3.75  1.89 | (1.12-22.9) ^*^  (1.04-13.6) ^*^  (0.34-3.35) |  | 0.73  0.72  0.56  0.29 | 1  1.16  1.24  1.45 | (0.51-2.61)  (0.61-2.52)  (0.73-2.88) |
| Education  ~ high school  college ~ | 0.22 | 1  1.34 | (0.84-2.15) |  | 0.83 | 1  1.14 | (0.35-3.72) |  | 0.44 | 1  1.23 | (0.61-2.52) |
| Sex  Male | 0.30 | 1.21 | (0.78-1.86) |  | 0.67 | 1.31 | (0.38-4.47) |  | 0.21 | 1.47 | (0.81-2.69) |
| Modified HAQ  Score=0 (no disability) | 0.01 | 1.80 | (1.18-2.74) ^*^ |  | 0.99 | 0.99 | (0.35-3.72) |  | <0.01 | 1.96 | (1.21-3.16) ^*^ |
| Glucocorticoid use  No | 0.05 | 1.58 | (1.01-2.47) ^*^ |  | 0.53 | 1.74 | (0.31-9.74) |  | 0.12 | 1.48 | (0.90-2.41) |
| Charlson comorbidity index  No | 0.39 | 1.21 | (0.78-1.87) |  | 0.07 | 3.26 | (0.93-11.5) |  | 0.78 | 1.07 | (0.67-3.06) |
| Rheumatoid factor  Negative | 0.44 | 1.27 | (0.69-2.35) |  | 0.69 | 0.81 | (0.28-2.36) |  | 0.41 | 1.39 | (0.63-3.06) |

**^*^**P<0.05; BRASS, Brigham Rheumatoid Arthritis Sequential Study; KORONA, KORean Observational study Network for Arthritis; OR, odds ratio; CI, 95% confidence interval; HAQ, health assessment questionnaire
